# Bibliometric profile of the African Academy of Sciences medical and health sciences fellows

**DOI:** 10.11604/pamj.2021.38.60.21004

**Published:** 2021-01-19

**Authors:** Joseph Balogun, Efe Mamuzo, Friday Okonofua, Adetutu Balogun, Adetoyeje Oyeyemi

**Affiliations:** 1 Department of Health Studies, Chicago State University, Chicago, USA,; 2 Centre of Excellence in Reproductive Health Innovation, University of Benin, Benin City, Edo State, Nigeria,; 3 University of Medical Sciences, Ondo City, Nigeria,; 4 Joseph Rehabilitation Center, Tinley Park, IL 60477, USA,; 5 Department of Physiotherapy, University of Maiduguri, Maiduguri, Borno State, Nigeria

**Keywords:** Scientometrics, bibliometric parameters, H-index, citations, co-authorship, fellowship, African Academy of Sciences, Google Scholar, Web of Science and Scopus databases

## Abstract

The African Academy of Sciences (AAS) is the preeminent science academy on the African continent, but there is currently no information on the academic productivity of the fellowship members. This study investigated the bibliometric parameters of the AAS medical and health sciences fellows. The demographic information (year of induction, gender, and region of employment in Africa) of the 80 medical and health sciences fellows were obtained from the AAS website. Subsequently, the bibliometric information (total number of publications, H-index scores, citation, and co-authorship counts) were extracted from the Scopus database. The majority of the fellows were from the East (36%) and West (33%) African regions (χ^2^ = p < 0.001); the North (6%) and Central (4%) regions were vastly underrepresented. Although only 34% of the AAS fellows were women, there was no statistically significant difference (p > 0.05) in the bibliometric parameters of both genders. The year of induction as a fellow and region of employment in Africa significantly (p < 0.05) influenced the bibliometric parameters. For all the fellows combined, their H-index mean (SD) score is 27.9 (17.0), while the median score for the total number of publications is 100, H-index is 27.5, and the citation and co-authorship count is 2,894 and 446, respectively. The fellows from the West African region had the highest number of publications (Mean = 212), citations (Mean = 9,437), and co-authorship count (Mean = 975), and the South African fellows had the highest H-index score (Mean = 40.8). The data presented provide insight into the bibliometric productivity of African scientists compared with their peers from other science academies around the world. Similarly, the data may assist burgeoning scientists aspiring to be AAS fellow set realistic goals toward achieving the stipulated H-index benchmarks.

## Introduction

The African Academy of Sciences (AAS) is the flagship science academy on the African continent with the mission to use science and technology to transform lives and pursue excellence by identifying and recognizing deserving scholars through the election of fellows [1]. In medicine and allied health professions, the fellowship is a mark of distinction representing the pinnacle of honor in professionalism, competence, and academic achievements [2]. The fellows of the AAS represents the most talented African scientists from 59 countries across the globe. It is surmised that AAS fellows have contributed significantly to the knowledge base in their disciplines, but there is presently no bibliometric data to support this speculation.

Bibliometrics is a discipline that uses objective measures to evaluate academic productivity. The field is changing rapidly with the development of new assessment tools, parameters, and normative data, but its use in health sciences is still in the developmental stage [3]. The novel measures for judging the productivity of scientists is the total number of publications, citation counts, and the H-index first conceptualized by Hirsch in 2005 [4]. Of all the bibliometric measures, the H-index is the most widely used measure of academic productivity. Based on its global appeal, Harzing conjectured that “unless you have been hiding under a stone in the last ten years, you will probably have heard about the h-index” [5].

Bibliometrics are presently used as the yardstick for judging the scientific and technological advancement of nations; countries with low research productivity remain underdeveloped [6-8]. Even in developing countries, the use of bibliometric measures is gradually gaining full acceptance. Two previous studies from Brazil published in 2008 [9, 10], investigated the H-index of the Brazilian Academy of Sciences and one of the studies compared the H-index of the Brazilian with the National Academy of Sciences of the USA [10]. The AAS in 2019 required scientists applying for fellowship to have a Scopus H-index score of 20 or higher with at least one of the scientists' journal article has 100 or more citations [1]. In 2019, the Nigerian National Universities Commission also mandated the use of Google Scholar's (H-index, and i10-index) in addition to other less objective criteria for promotion to full professorship. Similarly, the Nigerian Academy of Science has proposed the use of Google Scholar's H-index as one of the requirements for fellowship [11].

The medical and health sciences fellows of the AAS are highly respected power players in the health sectors of their respective countries. They engage with government officials and policymakers to foster the scientific and medical advancement of the African continent [1]. Paradoxically, there is currently no information in the literature on their academic productivity. Thus, upcoming academic talents do not have a reference benchmark against which to gauge their academic performance. This study was designed to evaluate the bibliometric parameters of the medical and health sciences fellows of the AAS. We hypothesized that the bibliometric parameters of the AAS fellows would be influenced by year of induction into fellowship, gender, and region of employment in Africa.

## Methods

**Sample:** bibliometric data on all the 80 medical and health sciences fellows of the AAS were collected during the study. The data for the associates, affiliates, and honorary fellows of the AAS were excluded. The data collection process began on September 26, 2019 and concluded on October 15, 2019.

**Procedures:** we accessed the website of the AAS and identified the list for the medical and health sciences fellows [1]. For each fellow, we obtained their names, the year of induction into the academy, gender, and region of employment in Africa. Subsequently, we extracted their bibliometric information (total number of publications or documents, H-index score, the total number of citations, and the number of co-authors) from the Scopus database [12]. We selected the Scopus platform for two main reasons. First, the Scopus H-index and citation scores are among the information now required for membership in the AAS. Second, the Scopus database has the most extensive collection of peer-reviewed literature of abstracts and citations than the other databases such as *ResearchGate, Google Scholar, Mendeley, Academia, and the Thomson-Reuter Institute of Scientific Information/Web of Science* [11]. On the other hand, Google Scholar has the best ranking of books and conference proceedings [13].

**Variables and measures:** the dependent variables in the study are the total number of publications, H-index, the total number of citations, and co-authorship counts. Gender, year of induction as a Fellow, and the country of employment in Africa (region) are the independent variables. The H-index is a robust single-number metric of a scholar's productivity and citation impact of the publications, combining quality with quantity. It reflects the scholar's most cited papers and the number of times other authors have referenced them. The H-index serves as an alternative to more traditional journal impact factor metrics used in the evaluation of the impact of the work of a particular researcher [14].

The H-index is calculated from the number of publications that other scientists have cited a scientist at least that same number of times. For example, an H-index of 17 indicates that the scientist has published at least 17 papers that have each been cited at least 17 occasions. If the scientist's 18^th^ most cited publication were cited only ten times, the H-index would remain at 17. If the scientist's 18^th^ most cited publication were cited 18 or more times, the H-index would rise to 18. On the other hand, the i10-index of a scientist is the number of publications with at least ten citations. The total number of publications, citations, and co-authorships provides additional bibliometric information on scientists' scholastic productivity. Only the Scopus database contains the co-authorship informatics.

**Ethical approval and sample:** the research protocol for this investigation was reviewed and approved (Reference # UMTH/REC/19/489) by the Research and Ethical Committee at the University of Maiduguri, Nigeria.

**Data analysis:** the data collected were coded and entered into the SPSS software database. The countries represented were Nigeria, Kenya, Ethiopia, South Africa, Morocco, Rwanda, Congo, Gambia, Mali, Tanzania, Senegal, Egypt, Sudan, Burkina Faso, Uganda, Ghana, Malawi, Zimbabwe, Cameroon, Madagascar, and Zambia. We categorized the country of employment of the fellows into five regions: North, West, East, Central, and South Africa [15]. Also, we classified the induction year into three groups: 2010 to 2019 represents group one; 2000-2009 belong to group two, and those elected before 2000 made up group three. We double-checked the raw data to ensure no error was made during data entry. Likewise, we evaluated the data set for the presence of outliers, normality, and homogeneity of variance to determine whether they met the assumptions for parametric tests. Based on the outliers on the boxplot and the result of the Shapiro Wilks and Levene's tests, we inferred that the data set did not meet the underlying assumptions guiding the use of the parametric analysis. Therefore, we evaluated differences between the groups using the nonparametric inferential tests (Mann Whitney U, Median, and Kruskal Wallis). The significant level was fixed at an alpha level of 0.05. We calculated the mean and median descriptive statistics to allow a robust comparison of our h-indexes with those from previous studies.

## Results

**Demographic data:** the demographic profile of the study participants is presented in Table 1. The year of induction of the fellows ranged from 1986 to 2018. The majority (70%) of the sample were inducted as a fellow of the AAS within the last decade (X^2^ = 48.5, p < 0.001). Women were underrepresented in the academy that is 66% men (X^2^ = 8.5, p < 0.01). The majority of the AAS members came from East (36%) and West (33%) African regions (X^2^ = 35.0, p < 0.001). The North (6%) and Central (4%) regions were vastly underrepresented.

**Table 1:** demographic profile of the study participants

Independent Variables	Frequency	Percentage (%)	χ^2^
**Year of induction into fellowship**			
2010 - 2019	56	70	
2000 - 2009	11	14	48.5†
1980 - 1999	13	16	
**Gender**			
Male	53	66	8.5‡
Female	27	34	
**Region of employment in Africa**			
North Africa	5	6	
East Africa	29	36	
Central Africa	3	4	35.0†
West Africa	26	33	
South Africa	17	21	

‡ p <0.05; † p<0.001

**Bibliometric profile**: Table 2 presents the H-index, the total number of publications, citation, and co-authorship counts for the medical and health sciences fellows of the AAS. The measures of dispersion (range and standard deviation (SD) values) for the bibliometric parameters showed wide variability that is suggestive of a heterogeneous group. The mean (SD) of the number of publications and H-index for the fellows of the AAS is 129 and 27.9 (17.0), respectively. The mean number of citations is 5,540 and 800 for co-authorships. Based on the total number of publications and citations (Table 2), we inferred that the AAS medical and health sciences fellows have moderately high publication and citation impact output but comparatively low h-indexes.

**Table 2:** bibliometric parameters of the medical and health sciences scholars in the African Academy of Sciences

Measures of central tendency and variability								
Publication attributes	Min.	Max.	Range	Mean	SD	95% CI†	Median	Interquartile range
Total number of publications	1	587	586	129	113	104 - 154	100	103
H-index	1	71	70	27.9	17.0	24.1 - 31.7	27.5	23
Total number of citations	4	52,843	52,839	5,540	7,735	3,819 - 7,261	2,894	6,398
Total number of co-authors	14	5,665	5,651	800	1,041	569 - 1,032	446	943

† Confidence interval

**Bibliometric reference percentile values:** the bibliometric referenced percentile values for the AAS fellows is presented in Table 3. The median score for the total number of publications is 100, median H-index is 27.5 and the median total number of citations and co-authorship count is 2,894 and 446, respectively.

**Table 3:** reference percentile values for the bibliometric parameters (n=80)

Dependent Variables	5th percentile score	10th percentile score	25th percentile score	50th percentile Median	75th percentile score	90th percentile score	95th percentile score
Total number of publications	3	25	52	100	154	307	386
H-index score	3.0	7.0	15.0	27.5	37.5	53.7	61.0
Total number of citations	147	204	1,118	2,894	7516	15,234	17,872
Total number of co-authors	27	38	146	446	1,088	1,809	3,365

**Group bibliometric parameters:** the results of the Mood´s Median test showed no statistically significant difference (p>.05) between the bibliometric parameters of the males and females (Table 4). On the other hand, a statistically significant difference (p < 0.05) was found among the region of employment in Africa and the year of induction. For example, the AAS fellows from West Africa had the highest number of publications (Mean = 212) compared to the other regions (Table 5). Central African fellows had the least amount of publications (Mean = 72). South African fellows had the highest H-index score (40.8), and Central Africa had the lowest (18.7). Similarly, West African fellows had the highest number of citations (9,437), and co-authors (975) and Central Africa had the least citations (1,709), and co-authors (360). The mean bibliometric parameters of the AAS fellows in Group 1 (2010 - 2019) are consistently higher than those in Groups 2 (2000 - 2009) and Groups 3 (1980 - 1999). Group 3 had the lowest bibliometric parameters (Table 5).

**Table 4:** group comparison with Mood´s median test

Independent/dependent variables	χ^2^	df	Significance level
**Gender**			
Total number of publications	0.03	1	0.873
H-index score	0.02	1	0.878
Total number of citations	0.22	1	0.636
Total number of co-authors	0.89	1	0.344
**Year of induction as a fellow**			
Total number of publications	12.66	2	0.002
H-index score	18.62	2	0.001
Total number of citations	21.06	2	0.001
Total number of co-authors	16.15	2	0.001
**Region of employment in Africa**			
Total number of publications	9.95	4	0.043
H-index score	16.3	4	0.003
Total number of citations	11.7	4	0.019
Total number of co-authors	7.4	4	0.118

**Table 5:** group mean bibliometric parameter for gender, the region of employment in Africa and co-authorship

Independent variables	Total number of publications	H-index score M(SD)	Total number of citations	Total number of co-authors
**Gender**				
Males (n=53)	134	27.5 (19.1)	5,754	786
Females(n=27)	119	28.8 (12.2)	5,120	828
**Region of employment in Africa**				
North Africa (n=5)	94	21.0 (12.9)	3,402	909
East Africa (n=29)	101	25.3(15.9)	4,068	716
Central Africa (n=3)	72	18.7 (12.1)	1,709	360
West Africa (n=26)	212	24.8 (16.5)	9,437	975
South Africa (n=17)	129	40.8 (16.2)	5,540	800
**Year of induction into fellowship**				
2010 - 2019 (n=56)	158	33.8 (16.0)	1,855	1,055
2000 - 2009 (n=11)	79	19.4 (11.7)	739	312
1980 - 1999(n=13)	44	9.9 (5.3)	679	116

**A priori comparison of the H-index values of science academy scholars:** the *Web of Science* median H-index of the Brazilian biomedical science professionals was 22 while their peers from the USA was 66. The H-index value for the Brazilians is substantially lower when compared to the Americans (Table 6). Another study from Brazil also reported a *Web of Science* means H-index of 23 for the biomedical scientists. The Scopus median H-index for the Bosnia and Herzegovina Academy was eight with a mean score of 13. In our study, the *Scopus*median H-index was 27.5, and a mean of 27.9 (17.0). The H-indexes in our study are higher than the values previously reported for scientists from Brazil and Bosnia and Herzegovina academies, but lower than the USA scientists (Table 6).

**Table 6:** comparison of the H-index in this study with other science academies

S/N	Study	Country	Median score	Mean score
1	Mugnaini et al. Comparison of scientists of the Brazilian Academy of Sciences and of the National Academy of Sciences of the USA on the basis of the h-index. Braz J Med Biol Res, April 2008, Volume 41(4) 258-**262** http://www.scielo.br/scielo.php?script=sci_arttext&pid=S0100-879X2008000400001	Brazil USA	22 66	- -
2	**Kellner and PoncianoI**. H**-**index in the Brazilian Academy of Sciences - comments and concerns. An. Acad. Bras. Ciênc. vol.80 no.4 Rio de Janeiro Dec. 2008 http://www.scielo.br/scielo.php?pid=S0001-37652008000400016&script=sci_arttext	Brazil	-	23
3	Masic I. Evaluation of the Medical Academic Community of Bosnia and Herzegovina Based on Scopus Parameters. Med Arch. 2017 Jun;71(3):164-168. doi: 10.5455/ medarh. 2017.71.164-168	Bosnia and Herzegovina	-	12*
4	This study	Africa	28	31

* Calculated from the individual data presented in the article

## Discussion

This study sets out to evaluate the bibliometric parameters of the AAS medical and health sciences fellows. Using combination key words of H-index, publication, and citation, we conducted an exhaustive search of the literature on the PUBMED and CINAHL databases to ascertain the academic productivity of African scientists. Our search produced 27 “hits,” but none of the publications is from the African continent. This investigation is the first to document the total number of publications, H-index, the total number of citations, and the co-authorship capacity of AAS fellows. Several bibliometric studies exist on the academic productivity and citation impact of scholars in science and technology disciplines, but data on scientists in medical and health sciences is limited. The Brazilian and American studies [9, 10] evaluated only the H-index, but our study presented other bibliometric parameters such as the publication productivity, citation impact, and co-authorship capacity of the AAS fellows. Our research is the first to document the co-authorship count of science academy scientists. Co-authorship capacity is one of the critical and well-documented measures of scientific collaboration. Almost every aspect of the scientific collaboration network can be reliably tracked by analyzing co-authorship networks by bibliometric methods [16].

The demographic data in our study revealed that women are grossly underrepresented. They represent only 34% of the study participants. The lack of gender diversity in the AAS is indicative of the gender composition in African universities. For example, in 1981, only 15.3% of Nigerian dental practitioners were female, but by 2000, the statistics increased steadily to 35.1%. Comparatively, the rate of female medical practitioners in 1981 was 15.0% but increased to only 19.0% by the end of 2000 [17]. Likewise, only 38% of Nigerian physiotherapy graduates were women, and 62% were males [18]. Although progress had occurred regarding gender equity, the educational gap remains in many developing countries where women are treated as second-class citizens. In many African countries, gender inequality in education exists from primary school to tertiary institutions in favor of males [17-20].

The Scopus H-index of the Bosnia and Herzegovina Academy scholars [21] ranged from two to 49, with a mean score of 12.The H-indexes that we reported in this study are much higher than the values from the Brazilian [9, 10], and Bosnia and Herzegovina [21] academies but lower than their USA peers [10]. Comparison of our H-indexes with other science academies allowed us to put the African scientists' academic productivity in a global context. In both the Kellner and Ponciano [9] and Mugnaini, Packer, Meneghini [10] studies, the H-index for the American and Brazilian scientists were obtained from the Web of Science database, while the Bosnia and Herzegovina Academy scholars [21] and our study were obtained from the Scopus database. Our inferences should be taken with a dose of caution for two reasons. First, the data collection occurred at different periods. For example, one of the studies from the Brazilian Academy of Sciences [10] was implemented in 2006, while the review of the Bosnia and Herzegovina Academies [21] was in 2016 - a decade apart. Thus, comparing the bibliometric data obtained a decade apart from the two Academies may not reflect the current realities in both countries. Second, each bibliometric database has different coverage biases, and it may be unrealistic to compare H-index from Scopus with the Web of Science [10, 11].

The H-index score for *Google Scholar* is typically higher than the value obtained for *Web of Science, Research Gate*; the Scopus usually has the lowest H-index [11]. Before 1996, Scopus has a limited publication coverage but better conference coverage. Google Scholar's conference coverage is the best with more journals, but like Scopus, it has limited publication coverage before 1990. The omission of conference proceedings in the databases is problematic for scholars in the computer science discipline, where conference proceeding is a critical part of their scholarship [13, 22]. The Web of Science database does not take into consideration published books, contribution to book chapter, and peer-reviewed conference proceedings [8, 9].

In 2017, Masic [23] evaluated the bibliometric parameters of the 121 founding members of the International Academy of Health Sciences Informatics. About 33.3% of the members were from Europe, 32.5% from North America, 16.6% from Asia, 7.5% from South America, 5.8% from Australia, and only 4.2% from Africa. The author plotted the Scopus and Google Scholar H-indexes and number of citations of the individual members from the different continents, but no empirical data was presented. Consequently, we could not compare our Scopus bibliometric data with theirs.

### Study implications and recommendations

To achieve gender parity in the science academies in Africa, both the government and the educational institutions in each country have a vital role to play. African universities should be mandated to develop strategies, action plans, and policies that address issues of gender inequality, particularly in the medical and health sciences discipline that are dominated by men. Academic departments should be made to set annual targets for equal gender representation at all levels (administrative and academic levels supervisor, senior management, and board leadership). Most importantly, the deans and head of departments must be held accountable in achieving the set targets. Besides, universities should offer scholarships and develop mentorship programs that pair junior women faculty with senior-level women role models. The mentorship will increase the visibility and the likelihood that junior faculty will ascend to leadership positions. In addition, universities should promote networking among peer female lecturers and provide value-added training and professional development opportunities across disciplines. These efforts will ensure that women have diverse experiences and clinical skills needed to succeed in the academy. Many African countries have national gender policy that commits to ensuring affirmative action, but over the years, representation of women remains below the 35% target set by regional and international organizations [19, 20].

Although bibliometric parameters are today globally embraced in higher education, there are critics of its use to gauge academic productivity because of the following reasons. Scopus and Web of Science do not index many journals in developing countries and many scientists in developing countries are not familiar with the *Scopus, Web of Science databases, and the Publish and Perish* software because the resources needed to access them are not readily available to them. Besides, scientists from developed countries tend to be more “nationalistic” in their citations, which can negatively affect the H-index of scientists from developing countries. Thus, many critics opined that it is unfair to compare the academic productivity of scientists from institutions with different infrastructures. The H-index of African scientists is generally lower compared to their peers in developed countries despite their high productivity [9, 10]. This schism is because the majority of the publication of African scientists appears in local journals that are often not indexed by *Scopus* and *Web of Science* databases. It is plausible that the total number of publications and citations instead of H-index will better capture the productivity of scientists in developing countries.

The H-index has been criticized for been less affected by methodological articles proposing successful and impactful new techniques or therapies. Consequently, many journals with high impact factors are more concerned with review articles than original research investigations. The initial of some author's surname is influential in citation potential, and double-barreled names are at times incorrectly listed in many databases. Furthermore, the H-index does not take into consideration confounding factors such as multiple and “gratuitous” authorship. Since the total number of publications determines the H-index, young scientists are inherently at a disadvantage because of their short career, regardless of the impact of their work. In recognition of the above criticisms, new variants of H-index (g-index, m-quotient, hc-index, e-index, g-index, and i-10 (i-n) have been proposed [3, 5], but they are currently not used on a larger scale.

To address these concerns, the *Scopus* and *Web of Science* databases must henceforth index books and peer-reviewed conference proceedings. This expansion will be a step forward in capturing the academic productivity of scientists in developing countries and scholars in the arts and humanities disciplines where scholarship communication avenues are primarily books and conference proceedings. For African scientists to improve their bibliometric productivity, they would need to publish their work in journals that are indexed by the major online databases. Furthermore, the rigor of their research published in the local journals should be improved and the journals strengthened to publish more editions each year. This development will raise the standard of the African journals to the next level of excellence that will make their contents attractive for the major bibliometric databases to index them. In the interim, adequate funding by government is needed to enhance the overall development of university-based journals.

### Limitations of the study

The disciplines within the AAS represents ten clusters. Our investigation is limited to the medical and health sciences cluster. Follow-up studies should explore the bibliometric parameters of the other nine AAS clusters and compare the quality, impact, and sustainability of their productivity with their medical and health sciences fellows and scientists from the other academies around the world.

The bibliometric profile of the AAS fellows in our study showed wide variability. For example, the lowest H-index score was one and the highest was 71. The wide variability in the data for all the bibliometric parameters is reflective of the fact that the selection criteria for fellowship is based on both academic (number of publication and impact, innovations), and service (leadership roles, and contribution to policy) achievements. The fellows inducted based on service criterion have limited productivity in the scholarship domain.

## Conclusion

Our findings revealed that women are underrepresented within the AAS. The fellows from the West African region had the highest number of publications (Mean=212), citations (Mean=9,437), and co-authorship counts (Mean = 975). On the other hand, South African fellows had the highest H-index score (40.8). The H-indexes in this study is higher than the values previously reported for Brazilian and Bosnia and Herzegovina science academies, but lower than the USA science academy. The data presented in this study will enhance the comparison of the bibliometric productivity of African scientists with their peers from other science academies around the world. Similarly, the data may assist burgeoning scientists aspiring to be AAS fellow set realistic goals toward achieving the stipulated H-index benchmarks.
